# Coracoid bone‐block versus soft‐tissue stabilization for anterior shoulder instability in professional athletes: A systematic review and meta‐analysis of return to play and recurrence rates

**DOI:** 10.1002/jeo2.70819

**Published:** 2026-06-22

**Authors:** Riccardo D'Ambrosi, Katia Corona, Angelo De Crescenzo, Valentina Fogliata, Valentina Greco, Andrea Pautasso, Andrea Sessa, Enrico Bellato, SICSeG (Italian Society of Shoulder, Elbow Surgery) Research Committee

**Affiliations:** ^1^ IRCCS Ospedale Galeazzi—Sant'Ambrogio Milan Italy; ^2^ Link Campus University Rome Italy; ^3^ Department of Medicine and Health Sciences “Vincenzo Tiberio” University of Molise Campobasso Italy; ^4^ Ente Ecclesiastico Ospedale Generale F. Miulli, Acquaviva delle Fonti Bari Italy; ^5^ UO Chirurgia della Spalla, Humanitas Gavazzeni e Humanitas Castelli Bergamo Italy; ^6^ ASST Ovest Milanese—Ospedale Nuovo di Legnano Legnano Italy; ^7^ ASST Sette Laghi—Ospedale di Circolo e Fondazione Macchi di Varese—Polo Universitario Varese Italy; ^8^ Shoulder and Elbow Unit Istituto Ortopedico Rizzoli Bologna Italy; ^9^ Department of Surgical Sciences, San Luigi Gonzaga Hospital University of Turin Orbassano Italy

**Keywords:** Bankart repair, Latarjet, meta‐analysis, professional athletes, return to play, shoulder instability

## Abstract

**Purpose:**

Anterior shoulder instability is a common injury among professional athletes. Both soft‐tissue and bone‐block procedures are widely used for surgical stabilization, but it remains unclear whether one approach offers superior outcomes in terms of return to play (RTP) and recurrence. The purpose of this systematic review and meta‐analysis was to compare RTP rates, time to RTP and recurrence of instability between professional athletes treated with coracoid bone‐block procedures and those undergoing soft‐tissue stabilization.

**Methods:**

A systematic search of PubMed, Embase and the Cochrane Library was conducted from database inception through August 2025 according to PRISMA (Preferred Reporting Items for Systematic Reviews and Meta‐Analyses) guidelines. Studies including professional athletes treated surgically for anterior shoulder instability were analysed. Pooled effect estimates were calculated using random‐effects models. Subgroup analyses compared bone‐block versus soft‐tissue stabilization for RTP, time to RTP and recurrence.

**Results:**

Thirteen studies were included in the systematic review and meta‐analysis and of these, eight analysed soft‐tissue surgery and five coracoid bone block. The overall pooled RTP rate was 95.6% (95% confidence interval [CI], 88.2–99.8) in the coracoid bone‐block group and 95.9% (95% CI, 91.6–98.9) in the soft‐tissue group (*p* = 0.781). No significant differences were found in the level of RTP between the two treatment groups (*p* = 0.266). A shorter time to RTP was observed in the coracoid bone‐block group (166.24 days [95% CI, 116.05–238.13]) compared with soft‐tissue stabilization (271.09 [95% CI, 195.03–376.80]; however, this finding should be interpreted with caution due to overlapping CIs, borderline statistical significance and substantial heterogeneity. The overall recurrence rate was 4.8% (95% CI, 1.5–9.3) with no difference between coracoid bone‐block procedure (2.6% [95% CI, 0.0–8.7]) and soft‐tissue (6.9% [95% CI, 2.0−13.8]) (*p* = 0.302).

**Conclusion:**

Both coracoid bone‐block and soft‐tissue procedures allow professional athletes to achieve high RTP rates with low recurrence. No definitive differences between techniques can be established, particularly considering the low quality of evidence and the substantial heterogeneity across studies.

**Level of Evidence:**

Level III, systematic review and meta‐analysis.

**Abbreviations**
CCT: controlled (non‐randomized) clinical trial
CI: confidence interval
MeSH: Medical Subject Headings
MINORS: Methodological Index for Nonrandomized Studies
PRISMA: Preferred Reporting Items for Systematic Reviews and Meta‐Analyses
RCT: randomized controlled trial
REML: restricted maximum‐likelihood
RTP: return to play
SD: standard deviation

## INTRODUCTION

Anterior shoulder instability is a common and clinically relevant condition among professional athletes, particularly in collision and overhead sports. The combination of repetitive microtrauma, high‐energy impacts and pressure for early return to competition places this population at increased risk of recurrent instability following both primary and surgical treatment. Consequently, the primary goal of management extends beyond restoring stability to enabling a rapid and safe return to play (RTP) at the preinjury level while minimizing long‐term functional impairment [9, 37].

Soft‐tissue stabilization procedures, most commonly arthroscopic Bankart repair with or without remplissage, have traditionally been considered the standard treatment for patients without significant bone loss. However, in professional athletes, these procedures have been associated with relatively high recurrence rates and suboptimal return to preinjury performance levels. These findings are likely related to the high biomechanical demands of elite sport, where early return to contact or overhead activity may exceed the biological healing capacity of repaired capsulolabral structures [4, 21].

Coracoid bone‐block procedures, including the Latarjet and Bristow techniques, have therefore gained increasing popularity in high‐risk populations. By addressing both capsulolabral insufficiency and glenoid bone loss, and by providing a dynamic sling effect, these procedures enhance anterior shoulder stability under high‐demand conditions. Recent technical advancements and expanding indications have led some authors to advocate bone‐block procedures as a primary stabilization strategy in elite athletes [3, 8, 12, 15, 16, 36].

Despite these considerations, direct comparisons between coracoid bone‐block and soft‐tissue stabilization in professional athletes remain limited. Existing studies are heterogeneous in terms of surgical technique, sport type and outcome definitions and most prior systematic reviews have included mixed populations of recreational and professional athletes, potentially limiting the applicability of their findings to elite cohorts [1, 24].

Therefore, the purpose of this systematic review and meta‐analysis was to compare return‐to‐sport rates, time to return to sport and recurrence of instability between professional athletes treated with coracoid bone‐block procedures and those undergoing soft‐tissue stabilization.

It was hypothesized that coracoid bone‐block procedures would be associated with a faster return to sport and lower recurrence rates compared with soft‐tissue stabilization, while achieving similar overall return‐to‐sport rates in professional athletes.

## MATERIAL AND METHODS

A systematic search strategy was developed according to the Preferred Reporting Items for Systematic Reviews and Meta‐Analyses (PRISMA) guidelines and is registered in the PROSPERO Registry (CRD420251118183) [25, 26].

An electronic database search was performed from database inception through 31 August 2025 to identify potentially relevant research articles that analysed RTP, time to RTP, level of RTP and percentage of new dislocation in elite and professional athletes after shoulder surgery for shoulder instability.

The search strategy included combinations of Medical Subject Headings (MeSH) and free‐text terms related to shoulder instability and surgical treatment, including ‘shoulder instability’, ‘shoulder dislocation’, ‘Bankart’, ‘Latarjet’, ‘Bristow’, ‘bone block’, ‘athlete’, ‘elite’ and ‘professional’, combined using appropriate Boolean operators (AND/OR). The full search strategies for each database are provided in the Supporting Information.

### Eligibility criteria

The literature selected for this study was selected on the basis of the following criteria.

### Study design

Randomized controlled trials (RCTs), controlled (non‐randomized) clinical trials (CCTs), prospective and retrospective comparative cohort studies, case–control studies and case series were included. Case reports and case series that did not report data on RTP, RTP level, time to RTP or the rate of new dislocation. RTP was defined as participation in at least one game in the same league as before the injury. Studies reporting on overlapping patient populations with the same outcomes were also excluded (only one such study was included in the review). Given the limited availability of high‐level evidence in professional athletes, a broad range of study designs, including non‐randomized studies and case series, was included to capture the existing literature on this specific population.

### Participants and interventions

Studies were conducted on skeletally mature elite or professional athletes treated for shoulder instability and evaluated for RTP activity, time to return, level of RTP and rate of new dislocation. All studies included professional athletes competing at national or international level, while mixed or recreational cohorts were excluded.

A minimum follow‐up of one full competitive season was required to ensure that RTP outcomes could be adequately assessed in a professional athletic context.

An elite athlete was defined as an individual participating in high‐level competitive sport at national or international level, including both professional athletes and academy‐level players involved in structured elite training programs. When studies included mixed cohorts, only data clearly referring to elite or professional‐level athletes were considered.

### Types of outcome measures

Four outcome measures were extracted and analysed:

- Return to play: defined as participation in at least one official match in the same sport after surgery.
- Time to return to play: defined as the time interval between surgery and the first official match, expressed in days.
- Return to preinjury level: defined as return to the same or higher level of competition compared with the preinjury status.
- Recurrent instability: defined as any new episode of ipsilateral shoulder dislocation or subluxation after surgery.

For the purpose of analysis, included studies were divided into two groups based on the surgical strategy:

a. Coracoid *bone‐block group*, comprising procedures involving coracoid transfer (Latarjet, Bristow or other autologous bone‐block techniques); and
b. *Soft‐tissue group*, including capsulolabral stabilization procedures such as Bankart repair, with or without remplissage or capsular shift.

## DATA COLLECTION AND ANALYSIS

### Study selection

The retrieved articles were first screened by title and, if relevant, further screened by reading the abstracts. After studies that did not meet the eligibility criteria were excluded, the entire content of the remaining articles was assessed for eligibility. To minimize the risk of bias, the authors reviewed and discussed all the selected articles, references and articles excluded from the study. In the case of any disagreements among the reviewers, the senior investigator made the final decision. At the end of the process, further studies that might have been missed were searched manually by going through the reference lists of the included studies and relevant systematic reviews.

### Data collection process

The data were extracted from the selected articles by the first two authors using a computerized tool created with Microsoft Access (Version 2010; Microsoft Corp). Each article was validated again by the first author before analysis. For each study, data regarding the patients were extracted (age, sex, sports practised), RTP, time to RTP, level of postoperative activity and new dislocation rate.

### Level of evidence

The Oxford Levels of Evidence set by the Oxford Centre for Evidence‐Based Medicine were used to categorize the level of evidence [6].

### Evaluation of the quality of studies

The quality of the selected studies was evaluated using the Methodological Index for Nonrandomized Studies (MINORS) score. The checklist includes 12 items, of which the last four are specific to comparative studies. Each item was given a score of 0–2 points. The ideal score was set at 16 points for noncomparative studies and 24 points for comparative studies [34].

### Statistical analysis

Meta‐analyses on the frequency of athletes who RTP to the same level and with new dislocation were conducted using a random‐effects model with the DerSimonian–Laird estimator for the variance. The raw proportions were stabilized using the Freeman–Tukey double arcsine transformation. The pooled estimates were presented as pooled proportions with corresponding 95% confidence intervals (CIs).

It was also performed a meta‐analysis on time to RTP with a random‐effect model on log transformed mean time using the restricted maximum‐likelihood (REML) estimator for variance. The pooled estimates were presented as pooled means with 95% CI. The meta‐analysis included primary studies with available standard deviation (SD) or range, from which we estimated the SD [38].

Differences among groups were explored with mixed‐effects meta‐regression models with common between‐study variance, using transformations and variance estimators previously described. For each outcome, within‐ and between‐group heterogeneity was assessed using Cochran's *Q* test. Group comparisons were performed with the ‘Soft‐tissue’ technique as reference category due to the highest number of studies in this group compared to coracoid bone‐block.

Between‐study variations were assessed for each model with the Cochran's *Q* test of heterogeneity and the Higgins *I* ^2^ statistic. Statistical heterogeneity was defined as substantial if *I* ^2^ > 50% [7]. Publication bias and small‐study effect were assessed through the funnel plot and doi plot. Funnel plot symmetry was tested with rank correlation test and the regression test, while the Luis Furuya‐Kanamori (LKF) index was calculated with the doi plot. A sensitivity analysis with the Trim‐and‐fill method was performed, and the fail‐safe *N* was calculated using the Rosenthal approach (Supporting Information S1: Appendix 2).

Two‐tailed tests were performed. A *p* value of <0.05 was considered to indicate statistical significance. The analysis was carried out using R (Version 4.3.0; R Foundation for Statistical Computing; URL: https://www.R-project.org/), specifically with meta (Version 8.0.1) and metafor packages (Version 4.2.0).

## RESULTS

**Figure 1:**
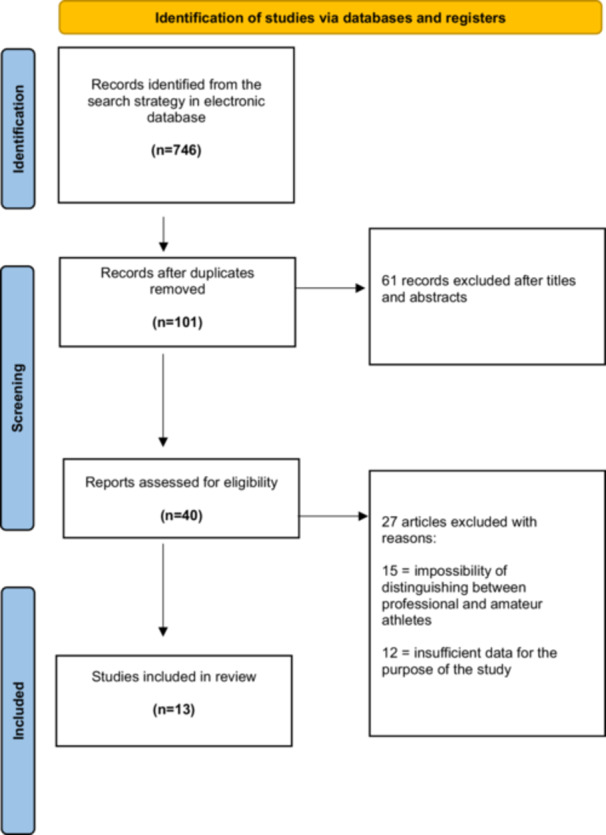
PRISMA (Preferred Reporting Items for Systematic Reviews and Meta‐Analyses) flow chart indicating research article inclusion for final analysis.

Initially, a thorough search of the three electronic databases yielded 746 records. The titles and abstracts of 101 studies were reviewed after removing 645 duplicates. After title and abstract screening, 61 studies were removed and 40 full‐text articles were assessed for eligibility. Finally, the reviewers excluded 27 records after assessing the full texts, and 13 articles were included in the final analysis of this review. The PRISMA diagram is shown in Figure 1.

Of these, eight analysed soft‐tissue surgery, and five coracoid bone block. Details of the studies are reported in Table 1.

**Table 1:** Characteristics of the studies included in the meta‐analysis.

Author	MINORS	Technique	Sports	Number of athletes	Mean age	Return to play	Level of return	Time to return (days)	New dislocation
Soft‐tissue									
Park et al. [27]	14	Arthroscopic Bankart	Baseball	51	20.9	42/51	41/51	252	NA
Paul et al. [29]	14	Arthroscopic Bankart	Baseball	64	23.0 ± 3.7	61/64	NA	257 ± 75.5 (37 athletes) 214 ± 84.9 (22 athletes) 286 ± 56.3 (5 athletes)	NA
Gibson et al. [11]	14	Arthroscopic Bankart	Soccer	34	23 (17−33)	34/34	NA	77 (63−98)	3/34
Domos et al. [10]	18	Arthroscopic Bankart (20 athletes)/arthroscopic Bankart and remplissage (20 athletes)	Collision athletes	40	25 (15−40)	38/40	38/40	91 (70−126)	7/40
Pavlik et al. [30]	12	Open Bankart	Different sports	35	23.3 (17−45)	34/35	23/35	279 (120−480)	0/35
Rangavajjula et al. [32]	12	Arthroscopic Bankart	Hockey	11	29 (20−36)	11/11	11/11	129 (57−219)	0/11
Kirac et al. [18]	20	Arthroscopic Bankart (34 athletes)/arthroscopic Bankart and remplissage (30 athletes)	Overhead athletes	64	34 athletes: 26.8 ± 4.9 30 athletes: 26 ± 5	NA	48/64	NA	3/64
Perret et al. [31]	18	Arthroscopic capsulolabral stabilization	Australian Football League	58	22.8 (18−33)	56/58	54/58	330.4 [median 207 (196–218)]	11/58
Coracoid bone‐block									
Bauer et al. [2]	14	Modified Latarjet	Handball	20	22.9 (17–35)	17/20	16/20	147 (105−210)	1/20
Brzoska et al. [5]	14	Arthroscopic Latarjet	Different sports	46	27.1 ± 7.3	44/46	40/46	150 ± 42	4/46
Kawasaki et al. [17]	12	Bristow	Rugby	176	18.9 (18.3–19.4)	NA	164/176	189 (174–204)	6/176
Perret et al. [31]	18	Open Latarjet	Australian Football	32	23.5 (13−30)	31/32	31/32	320.8 (241.6–400)	0/32
Stirma et al. [35]	12	Open Latarjet	Soccer	10	22.9 (16–28)	10/10	10/10	93.5 (60–120)	0/10

Abbreviations: MINORS, Methodological Index for Nonrandomized Studies; NA, not applicable.

**Figure 2:**
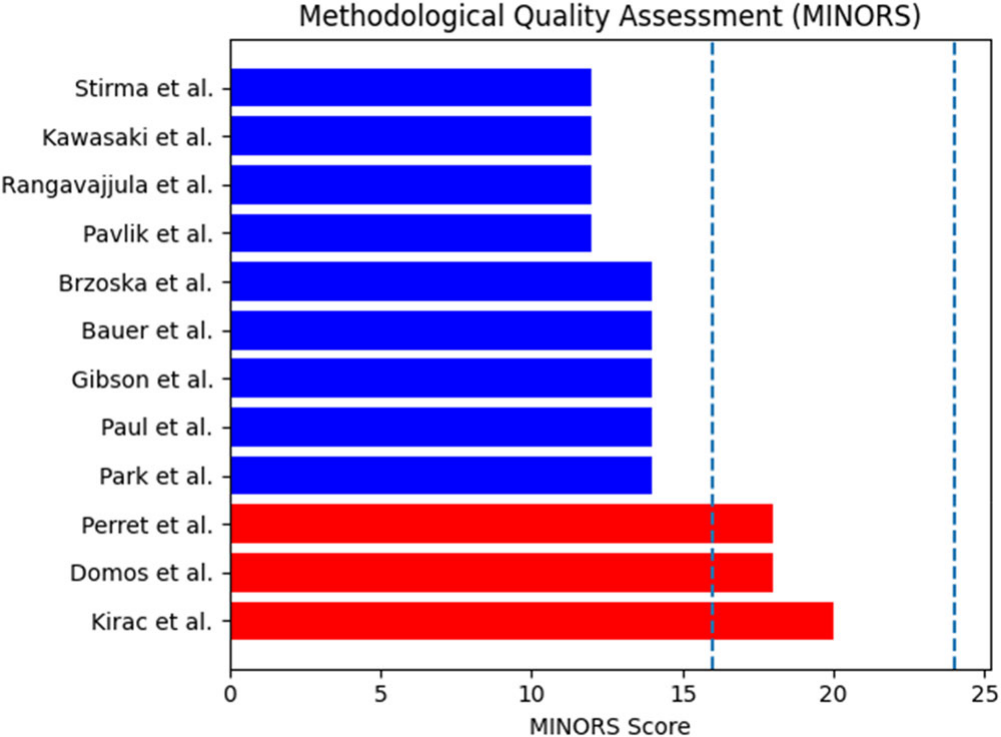
Methodological quality of included studies assessed using the MINORS score. Comparative studies (red; maximum score: 24) and non‐comparative studies (blue; maximum score: 16) are presented separately to account for differences in scoring systems. Dashed vertical lines indicate the maximum achievable scores for each study design. Overall, most studies demonstrated moderate methodological quality with variability across study types. MINORS, Methodological Index for Nonrandomized Studies.

The methodological quality of the included studies is summarized in Figure 2. Comparative studies showed higher absolute MINORS scores due to a greater number of evaluable items (maximum 24), whereas non‐comparative studies reached a maximum of 16 points.

Assessment of publication bias showed no evidence of significant asymmetry for most outcomes, as confirmed by funnel plot inspection and statistical tests. However, for time to RTP, evidence of asymmetry was detected, suggesting potential small‐study effects. Detailed results of these analyses are reported in Supporting Information S1: Appendix 2.

### Age

The pooled meta‐analysis showed no difference in terms of age at surgery between the two groups, with a mean estimated age of 23.69 years. Heterogeneity across studies was *I* ^2^ = 35.9% [0.0%; 68.5%].

### Return to play

The pooled meta‐analysis showed no statistically significant difference in the percentage of RTP between the coracoid bone‐block group (95.6% [95% CI, 88.2–99.8]) and the soft‐tissue group (95.9% [95% CI, 91.6–98.9]) (*p* = 0.781).

**Figure 3:**
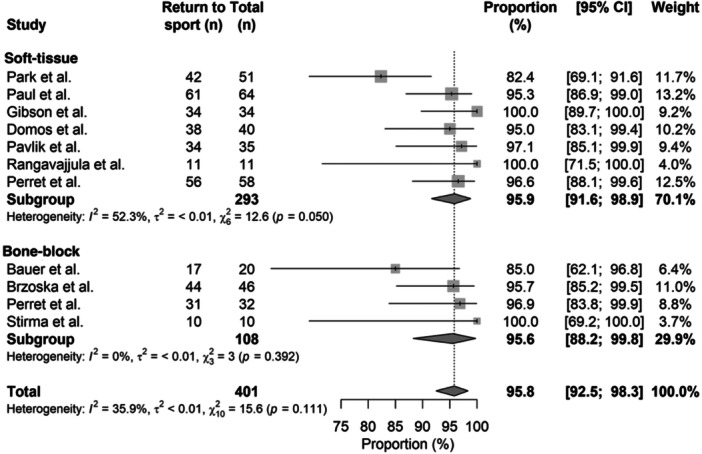
Forest plot of return to play between soft‐tissue surgery and bone‐block procedure. CI, confidence interval.

Heterogeneity across studies was *I* ^2 ^= 35.9%. The forest plot in Figure 3 shows details regarding percentage of RTP.

### Level of return

**Figure 4:**
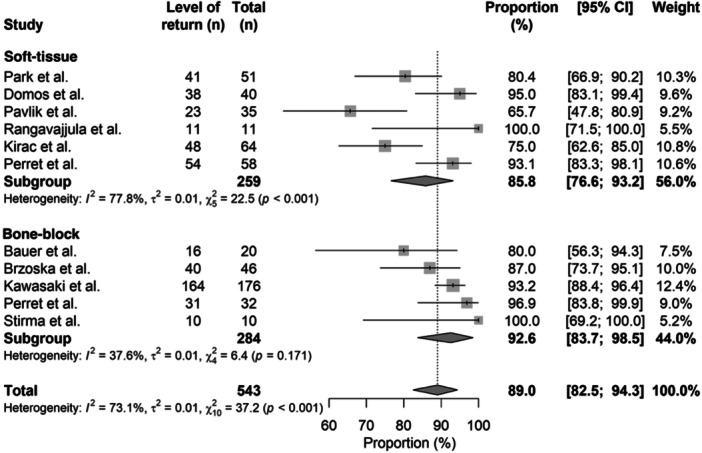
Forest plot of the level of RTP between soft‐tissue surgery and bone‐block procedure. CI, confidence interval; RTP, return to play.

The pooled meta‐analysis showed no difference regarding the level of return between the coracoid bone‐block group (92.6% [95% CI 83.7–98.5]) and the soft‐tissue group (85.8% [95% CI 76.6–93.2]) (*p* = 0.266). Heterogeneity across studies was *I* ^2 ^= 73.1%. The forest plot in Figure 4 shows details regarding level of RTP.

### Time to return

The pooled mean time to RTP was 271.09 days (95% CI, 195.03–376.80) in the soft‐tissue group and 166.24 days (95% CI, 116.05–238.13) in the coracoid bone‐block group. This corresponds to a descriptive difference of approximately 105 days. However, the CIs of the two groups partially overlapped, and the between‐group comparison showed only marginal statistical significance (*p* = 0.049) in the presence of extremely high heterogeneity (*I* ^2 ^= 100%).

**Figure 5:**
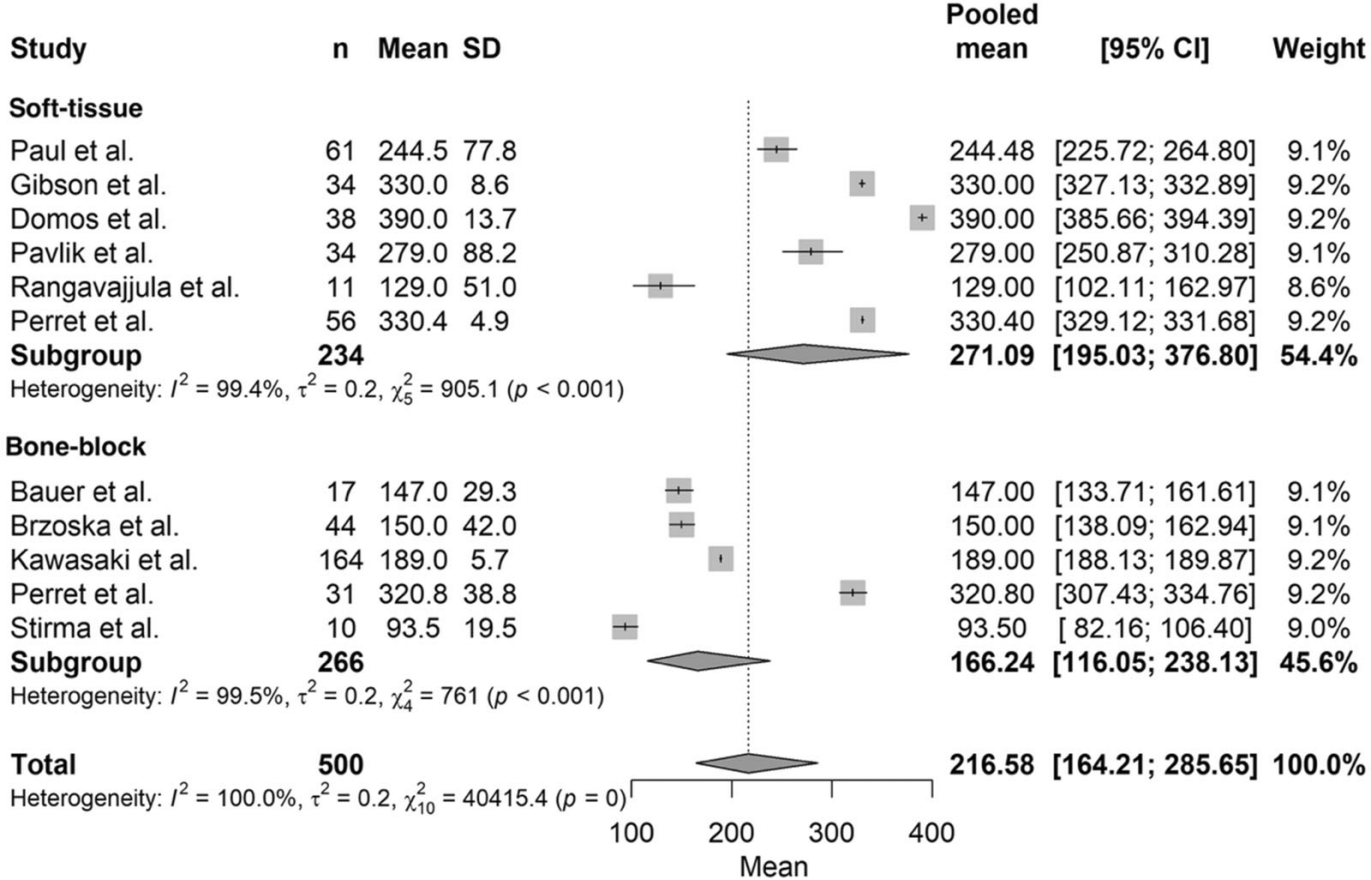
Forest plot of time to RTP between soft‐tissue surgery and bone‐block procedure. CI, confidence interval; RTP, return to play; SD, standard deviation.

The forest plot in Figure 5 shows details regarding time of RTP.

### New dislocation

**Figure 6:**
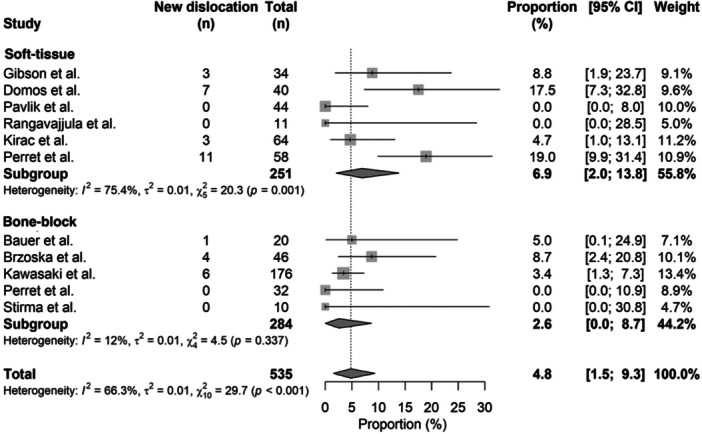
Forest plot of the new dislocation between soft‐tissue surgery and bone‐block procedure. CI, confidence interval.

The pooled meta‐analysis showed no difference regarding new dislocation episodes between the two groups (*p* = 0.302). Heterogeneity across studies was *I* ^2 ^= 66.3%. The forest plot in Figure 6 shows details regarding time of RTP.

## DISCUSSION

The principal finding of this systematic review and meta‐analysis is that both coracoid bone‐block and soft‐tissue stabilization procedures provide high rates of RTP and low recurrence in professional athletes with anterior shoulder instability. Although a shorter time to RTP was observed in the coracoid bone‐block group, this finding should be interpreted with caution. The CIs of the two groups overlapped, the statistical significance was marginal (*p* = 0.049), and heterogeneity was extremely high (*I* ^2 ^= 100%). Moreover, the presence of substantial asymmetry in the doi plot (LKF index: −6.91) suggests potential small‐study effects, further limiting the reliability of this result. Taken together, these findings suggest that time to RTP should not be considered a reliable differentiating factor between surgical techniques based on the current evidence.

RTP outcomes in professional athletes after anterior shoulder stabilization are a crucial metric of surgical success. In our pooled analysis, the rate of return to sport exceeded 90% in both groups, consistent with prior reports. Rossi et al. reported a 94% return to competition and 84% return to preinjury level among competitive athletes undergoing the open Latarjet procedure with less than 20% glenoid bone loss, with a recurrence rate of 4.6% at a mean 53‐month follow‐up [33] Similarly, Hurley et al. conducted a systematic review of 21 studies including 930 athletes and found RTP rates of 61%–94% after Bankart repair and 72%–96.8% after Latarjet, with no statistically significant difference in overall recurrence rates [13, 14]. However, the majority of these prior reviews pooled recreational, collegiate and professional cohorts. When focusing exclusively on professional athletes, our results suggest that subtle but meaningful differences emerge, particularly regarding time to return and the durability of shoulder stability during high‐intensity activity.

Our findings align with several sport‐specific series published in recent years. In professional rugby players, Kawasaki et al. reported a 93% return to preinjury level and a recurrence rate of 3.4% after the Bristow procedure, with a mean return time of 6.3 months [17]. Similarly, Mouchantaf et al. described 84% return and 9% recurrence following arthroscopic Latarjet in professional contact athletes [23]. Among professional handball players, Bauer et al. found 85% return and 80% at the same level at a mean of 4.9 months after open Latarjet–Patte [2]. In Australian Football League athletes, Perret et al. compared open Latarjet with soft‐tissue repair and found significantly fewer recurrences in the Latarjet group (0% vs. 19%) and a 97% RTP rate [31]. Even in non‐collision sports such as professional soccer, Stirma et al. reported 100% RTP within 3 months and no recurrence following open Latarjet [35].

The role of soft‐tissue stabilization should not be underestimated, as Bankart repair remains an effective option in selected athletes. Pasqualini et al. reported an 89% overall return and 73% same‐level return at 6 months after arthroscopic Bankart repair in professional soccer players, with only a 10% recurrence rate [28]. Similarly, Rangavajjula et al. observed that 80% of professional athletes treated arthroscopically for anterior instability returned to play, although recurrence rates reached 20% in contact sports [32]. These findings indicate that the main limitation of soft‐tissue repair in elite athletes lies not in the rate of return, which remains high, but in the durability of stability under competitive stress. Moreover, early RTP protocols after soft‐tissue repair (3–5 months) may expose the capsulolabral complex to repetitive load before full biological integration, explaining the higher recurrence observed in contact athletes [9]. The biomechanical margin of safety provided by coracoid bone augmentation may therefore justify its preference in elite collision sports or in cases requiring rapid reintegration into professional competition.

Although coracoid bone‐block procedures may provide enhanced stability, their potential complications must be carefully considered. These include graft‐related issues, neurovascular injury and the risk of long‐term degenerative changes. Therefore, despite their biomechanical advantages, these procedures should not be considered universally preferable, and appropriate patient selection, surgical expertise and postoperative management remain critical [13, 14, 19, 20, 22].

From a methodological perspective, this meta‐analysis has several strengths. It is the first to isolate professional athletes, eliminating the confounding effect of mixed cohorts. By stratifying procedures into soft‐tissue and coracoid bone‐block categories, the analysis provides clinically interpretable comparisons. The use of random‐effects models, heterogeneity assessment and publication bias evaluation ensures robustness of the pooled estimates.

However, several limitations should be acknowledged. First, substantial heterogeneity was observed across studies, likely related to differences in sport type, surgical technique, follow‐up duration and RTP definitions, which may limit the comparability of pooled estimates. Second, most included studies were retrospective Level IV investigations with moderate methodological quality, introducing potential selection bias, confounding by indication and variability in outcome assessment. In particular, patients with greater instability severity or bone loss were more likely to undergo coracoid bone‐block procedures, potentially influencing comparative outcomes. Additionally, sample sizes were generally small due to the rarity of elite athletic cohorts, and objective performance metrics were inconsistently reported. Variability in the definitions of ‘elite athlete’ and RTP outcomes may have further contributed to heterogeneity. Finally, long‐term outcomes and high‐quality prospective comparative studies remain limited, restricting the strength and durability of current evidence. Future studies should focus on prospective, sport‐specific cohorts with standardized definitions of RTP and consistent reporting of performance outcomes. Stratification by sport type and risk profile may further improve clinical decision‐making in this population.

From a clinical perspective, surgical management of anterior shoulder instability in professional athletes should be individualized. While coracoid bone‐block procedures may allow faster RTP, both techniques provide comparable overall return rates and recurrence outcomes. Therefore, treatment decisions should consider anatomical factors, sport‐specific demands and the athlete's performance goals.

## CONCLUSIONS

Both coracoid bone‐block and soft‐tissue procedures allow professional athletes to achieve high RTP rates with low recurrence. No definitive differences between techniques can be established, particularly considering the low quality of evidence and the substantial heterogeneity across studies.

## AUTHOR CONTRIBUTIONS

**Riccardo D'Ambrosi**: Conception; design; writing; materials; data collection; literature review. **Katia Corona**: Design; data collection; processing; analysis; writing; materials; literature review. **Angelo De Crescenzo**: Conception; supervision; interpretation; critical review. **Valentina Fogliata**: Writer; critical review; literature review; processing; interpretation; supervision. **Valentina Greco**: Supervision; literature review; materials; writer; critical review. **Andrea Pautasso**: Design; data collection; processing; analysis; writing; materials; literature review. **Andrea Sessa**: Literature review; materials; design; conception; analysis. **Enrico Bellato**: Conception; supervision; interpretation; critical review.

## FUNDING INFORMATION

Italian Ministry of Health—“Ricerca Corrente”.

## CONFLICT OF INTEREST STATEMENT

The authors declare no conflict of interest.

## ETHICS STATEMENT

The authors have nothing to report.

## Supporting information

Supporting File 1

## Data Availability

Raw data are available upon request to the corresponding author.
