# Supplementary material for: Coracoid bone‐block versus soft‐tissue stabilization for anterior shoulder instability in professional athletes: A systematic review and meta‐analysis of return to play and recurrence rates
Source: J Exp Orthop. 2026 Jun 22;13(2):e70819. doi: 10.1002/jeo2.70819 (PMC13285579; doi:10.1002/jeo2.70819)
Supplement: Supplementary file 1 — Supporting File 1 [file JEO2-13-e70819-s001.docx]

Appendix 1

Full search strategy

PubMed (MEDLINE):

("shoulder instability"[Title/Abstract] OR "shoulder dislocation"[Title/Abstract])

AND ("Latarjet"[Title/Abstract] OR "Bristow"[Title/Abstract] OR "Bankart"[Title/Abstract] OR "bone block"[Title/Abstract])

AND ("athlete"[Title/Abstract] OR "professional"[Title/Abstract] OR "elite"[Title/Abstract])

Embase:

('shoulder instability':ti,ab OR 'shoulder dislocation':ti,ab)

AND ('latarjet':ti,ab OR 'bristow':ti,ab OR 'bankart':ti,ab OR 'bone block':ti,ab)

AND ('athlete':ti,ab OR 'professional':ti,ab OR 'elite':ti,ab)

Cochrane Library:

("shoulder instability" OR "shoulder dislocation")

AND ("Latarjet" OR "Bristow" OR "Bankart" OR "bone block")

AND ("athlete" OR "professional" OR "elite")

Supplementary Material

Appendix 2

# Age

Mean age including the 13 studies: 23.7 (median: 23.0, IQR: 22.8-25.0, min-max:18.9-29.0).

# Meta-analysis: Return to Sport

Grafici: *1 Return to Sport*

## 2.1 Meta-analysis: overall

Number of studies: 11

Number of analysed patients: 401

Number of patients who returned to sport: 378

|  | Estimate [95%CI] |
| --- | --- |
| Pooled percentage (Random effects model) | 95.8 [92.5; 98.3] |
| **Heterogeneity** |  |
| tau^2^ | 0.004 [<0.001; 0.027] |
| I^2^ | 35.9% [0.0%; 68.5%] |

**Test of heterogeneity:** Q_10_= 15.6, p=0.111

## 2.2 Meta-analysis: By group

| **Characteristics of the model** | **Values** |
| --- | --- |
| tau^2^ | 0.005 (SE = 0.006) |
| R^2^ | 0.0% |
| **Test for subgroup differences** |  |
| Between group | Q_1_= 0.08, p-value=0.781 |
| Within group | Q_9_=15.6, p-value=0.077 |

**
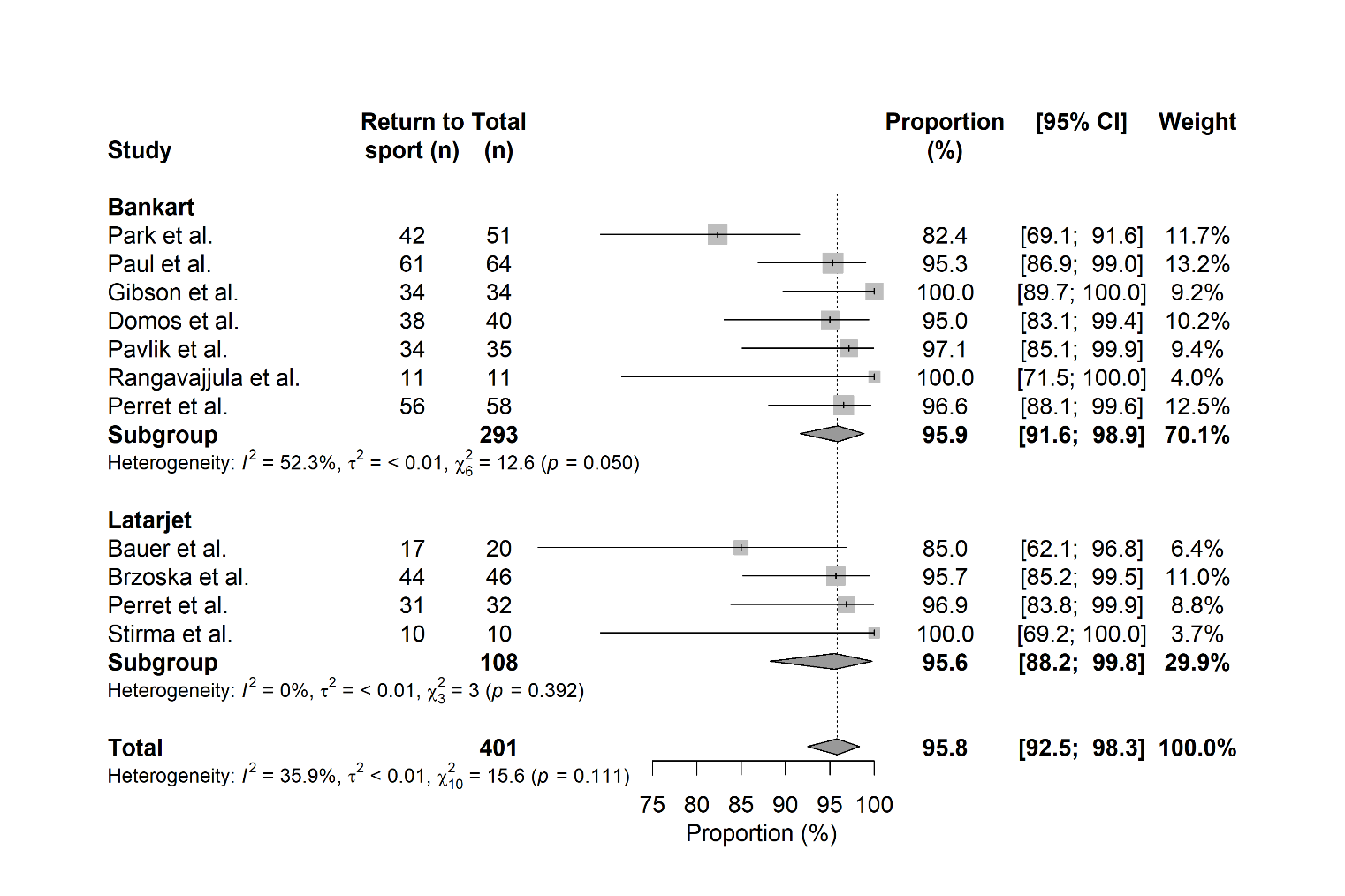
**


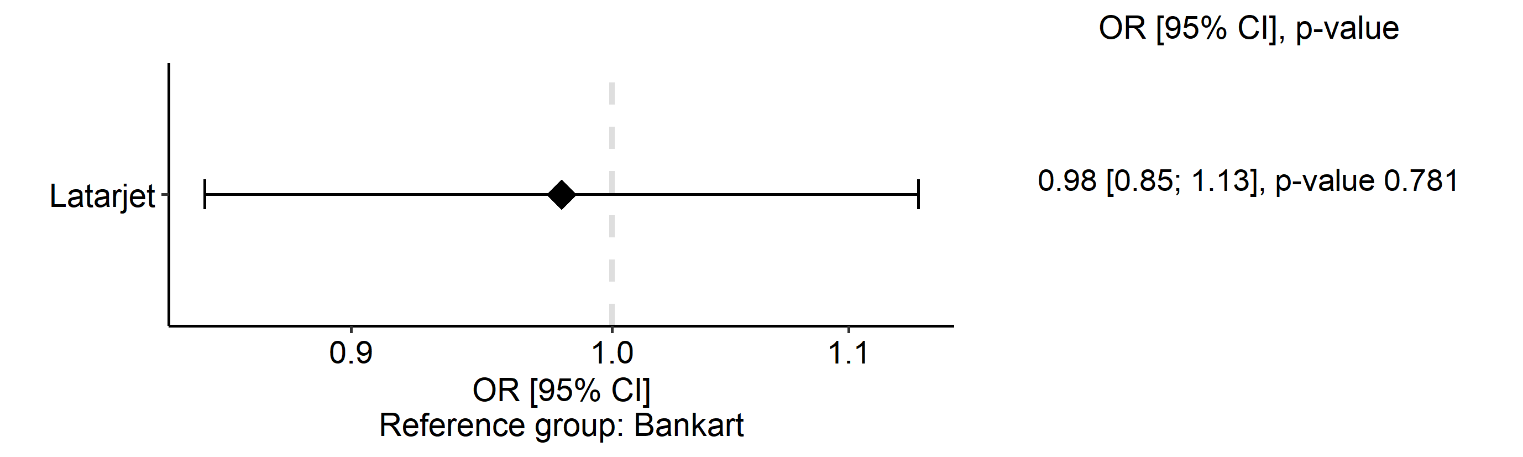


## 2.3 Funnel plot & doi plot

| Rank correlation test | p-value: 0.445 |
| --- | --- |
| Linear regression test | p-value: 0.579 |
| Doi plot | no asymmetry |
| LFK index | 0.41 |

## 2.4 Sensitivity analysis

- Overall estimate with Trim-and-fill method

Number of studies added: 3

Number of studies: 14

|  | Estimate [95%CI] |
| --- | --- |
| Pooled percentage | 94.8 [91.2; 97.6] |
| **Heterogeneity** |  |
| tau^2^ | 0.005 [<0.001; 0.027] |
| I^2^ | 41.3% [0.0%; 68.8%] |

**Test of heterogeneity:** p=0.053

- Fail-safe N Calculation Using the Rosenthal Approach

Fail-safe N: 10947

# Meta-analysis:: Level of Return

Grafici: *2 Level of Return*

## 3.1 Meta-analysis: overall

Number of studies: 11

Number of analysed patients: 543

Number of patients who returned to the same level: 476

|  | Estimate [95%CI] |
| --- | --- |
| Pooled percentage (Random effects model) | 89.0 [82.5; 94.3] |
| **Heterogeneity** |  |
| tau^2^ | 0.015 [0.004; 0.065] |
| I^2^ | 73.1% [50.8%; 85.3%] |

**Test of heterogeneity:** Q_10_= 37.2, p<0.001*

## 3.2 Meta-analysis: By group

| **Characteristics of the model** | **Values** |
| --- | --- |
| tau^2^ | 0.013 (SE = 0.010) |
| R^2^ | 10.1% |
| **Test for subgroup differences** |  |
| Between group | Q_1_=1.2, p-value=0.266 |
| Within group | Q_9_=28.9, p-value=0.001* |

**
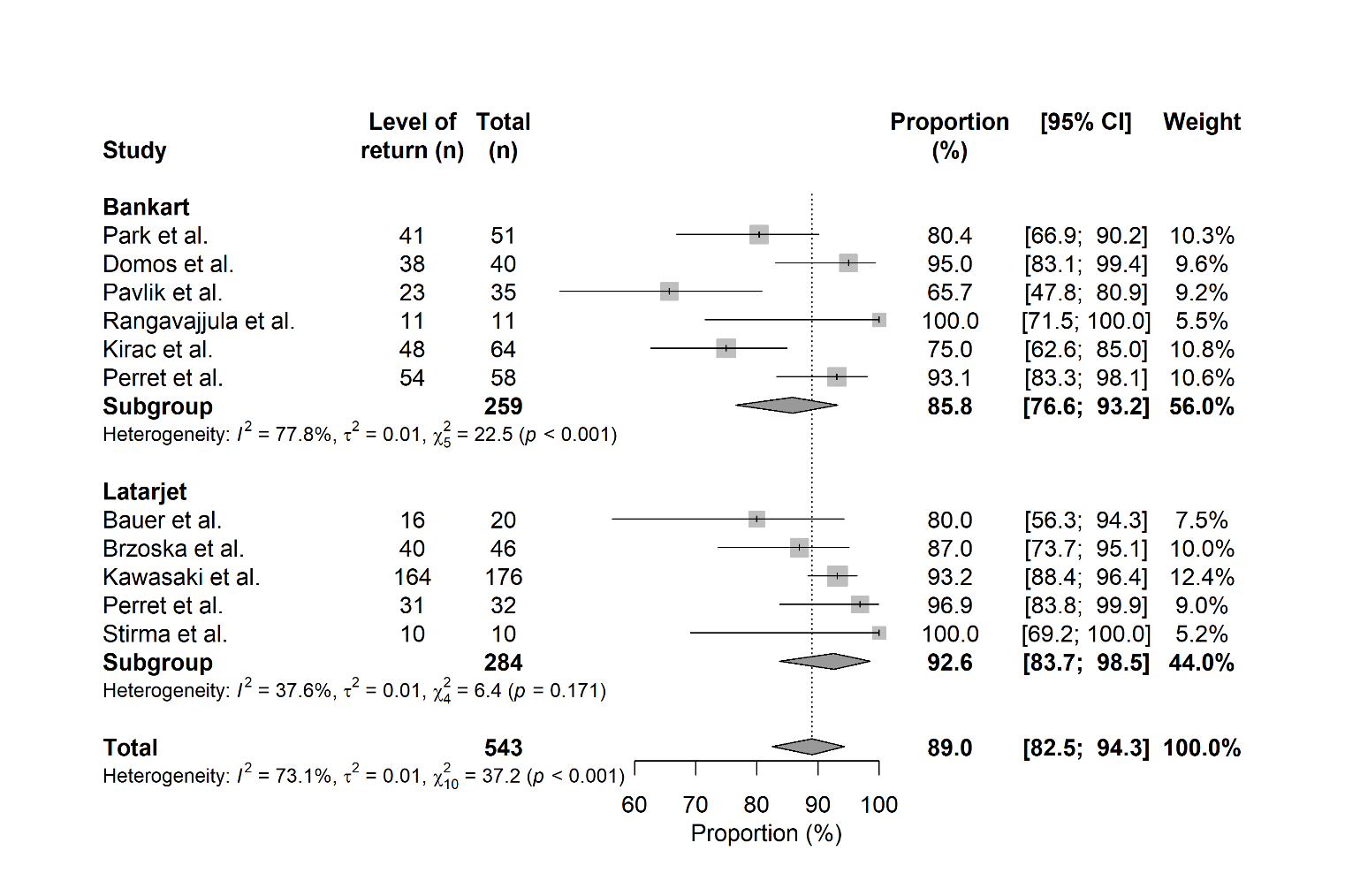
**


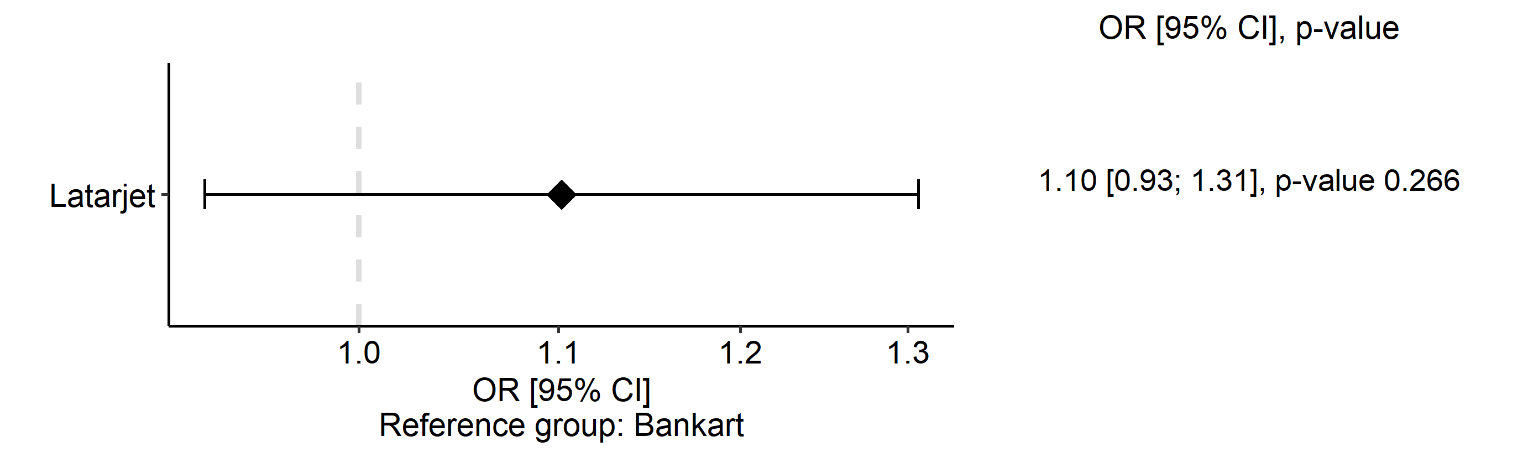


## 3.3 Funnel plot & doi plot

| Rank correlation test | p-value: 0.761 |
| --- | --- |
| Linear regression test | p-value: 0.394 |
| Doi plot | no asymmetry |
| LFK index | -0.9 |

## 3.4 Sensitivity analysis

- Overall estimate with Trim-and-fill method

Number of studies added: 0

Number of studies: 11

- Fail-safe N Calculation Using the Rosenthal Approach

Fail-safe N: 11366

# Meta-analysis:: Time to return (days)

Grafici: *3 time to return*

## 4.1 Mean time to return

Mean time to return to sport including the 12 studies providing the mean time among results (Time to return is missing in 1 study): 237.9 (median: 248.2, IQR: 149.2-323.1, min-max: 93.5-390.0).

## 4.2 Meta-analysis: overall

Number of studies: 11 (1 with missing time to return, 1 with missing SD)

Number of analysed patients: 500

|  | Estimate [95%CI] |
| --- | --- |
| Pooled mean (Random effects model) | 216.58 [164.21; 285.65] |
| **Heterogeneity** |  |
| Tau^2^ | 0.217 [0.105; 0.678] |
| I^2^ | 100.0% |

Test of heterogeneity: Q_10_=40415.4, p<0.001*

## Meta-analysis: by group

**
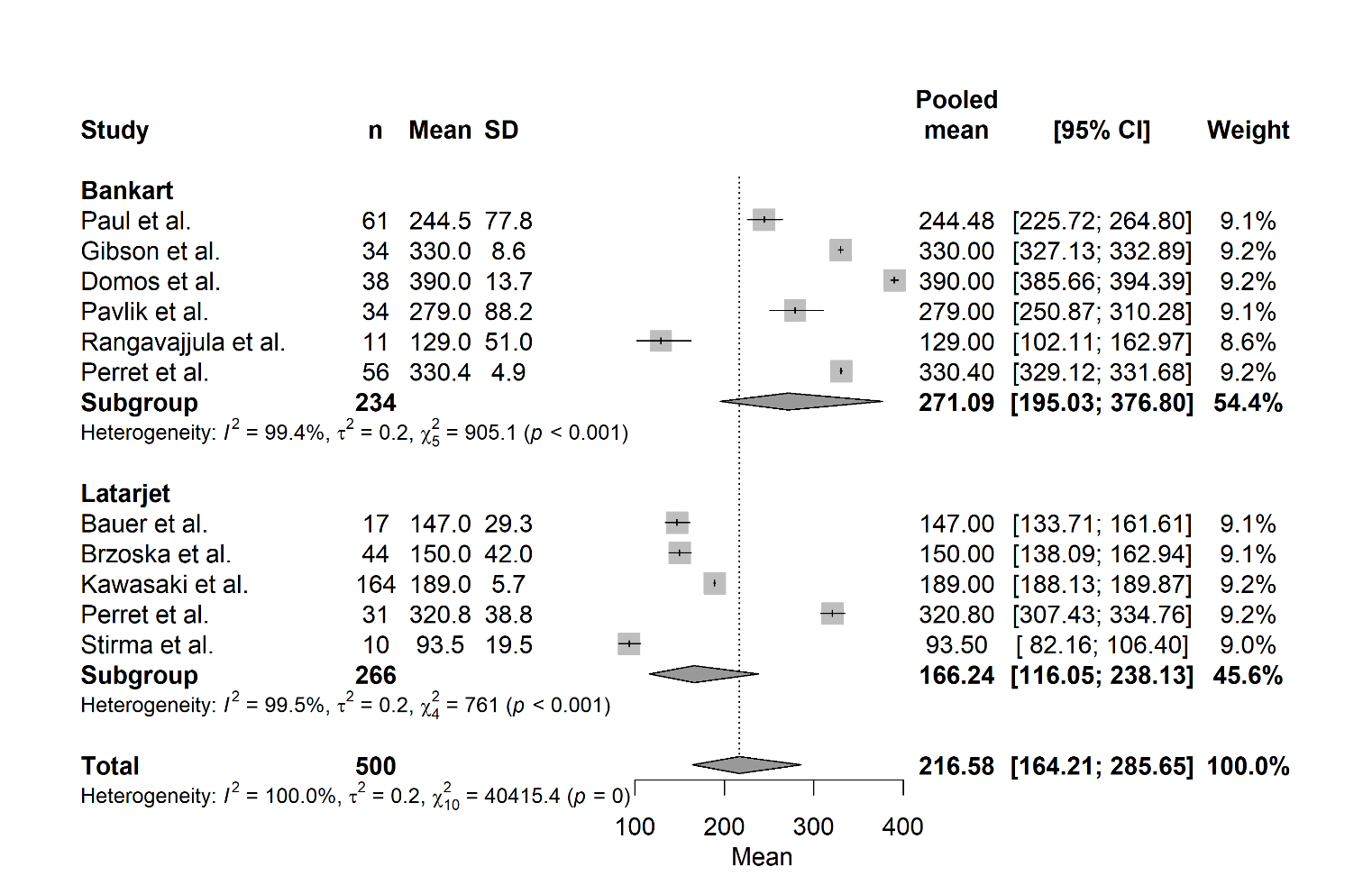
**

| **Characteristics of the model** | **Values** |
| --- | --- |
| Tau^2^ | 0.166 (SE = 0.080) |
| R^2^ | 25.3% |
| **Test for subgroup differences** |  |
| Between group | Q_1_=3.87, p-value:0.049* |
| Within group | Q_9_=1666.1, p-value<0.001* |

Estimates

|  | N of studies | Mean [95%CI] | Subgroup comparison  (p-value) |
| --- | --- | --- | --- |
| Bankart | 6 | 271.09 [195.03; 376.80] | Reference group |
| Latarjet | 5 | 166.24 [116.05; 238.13] | 0.049* |

## 4.4 Funnel plot & doi plot

| Rank correlation test | p-value: 0.121 |
| --- | --- |
| Linear regression test | p-value: 0.004* |
| Doi plot | Major asymmetry |
| LFK index | -6.91 |

**By group**

|  | Rank correlation test (p-value) | Egger’s Regression Test (p-value) | Doi plot asymmetry (interpretation) | LFK index |
| --- | --- | --- | --- | --- |
| Bankart | >0.99 | <0.001* | Major asymmetry | -6.38 |
| Latarjet | >0.99 | 0.054 | Major asymmetry | -4.59 |

## 4.5 Sensitivity analysis

- Overall estimate with Trim-and-fill method

Number of studies added: 3

Number of studies: 14

|  | Estimate [95%CI] |
| --- | --- |
| Pooled mean | 270.51 [196.10; 373.14] |
| **Heterogeneity** |  |
| tau^2^ | 0.374 [0.195; 0.985] |
| I^2^ | 100.0% |

**Test of heterogeneity:** p<0.001*

- Fail-safe N Calculation Using the Rosenthal Approach

Fail-safe N: 25811032

# Meta-analysis:: New Dislocation

Grafici: *4* *New Dislocation*

## 5.1 Metanalisi overall

Number of studies: 11

Number of analysed patients: 535

Number of patients with New Dislocation: 35

|  | Estimate [95%CI] |
| --- | --- |
| Pooled percentage (Random effects model) | 4.8 [1.5; 9.3] |
| **Heterogeneity** |  |
| tau^2^ | 0.011 [0.001; 0.047] |
| I^2^ | 66.3% [36.2%; 82.2%] |

**Test of heterogeneity:** Q_10_= 29.7, p=0.001*

## 5.2 Meta-analysis: By group

| **Characteristics of the model** | **Values** |
| --- | --- |
| tau^2^ | 0.011 (SE = 0.008) |
| R^2^ | 0.9% |
| **Test for subgroup differences** |  |
| Between group | Q_1_= 1.1, p-value=0.302 |
| Within group | Q_9_=24.9, p-value=0.003* |

**
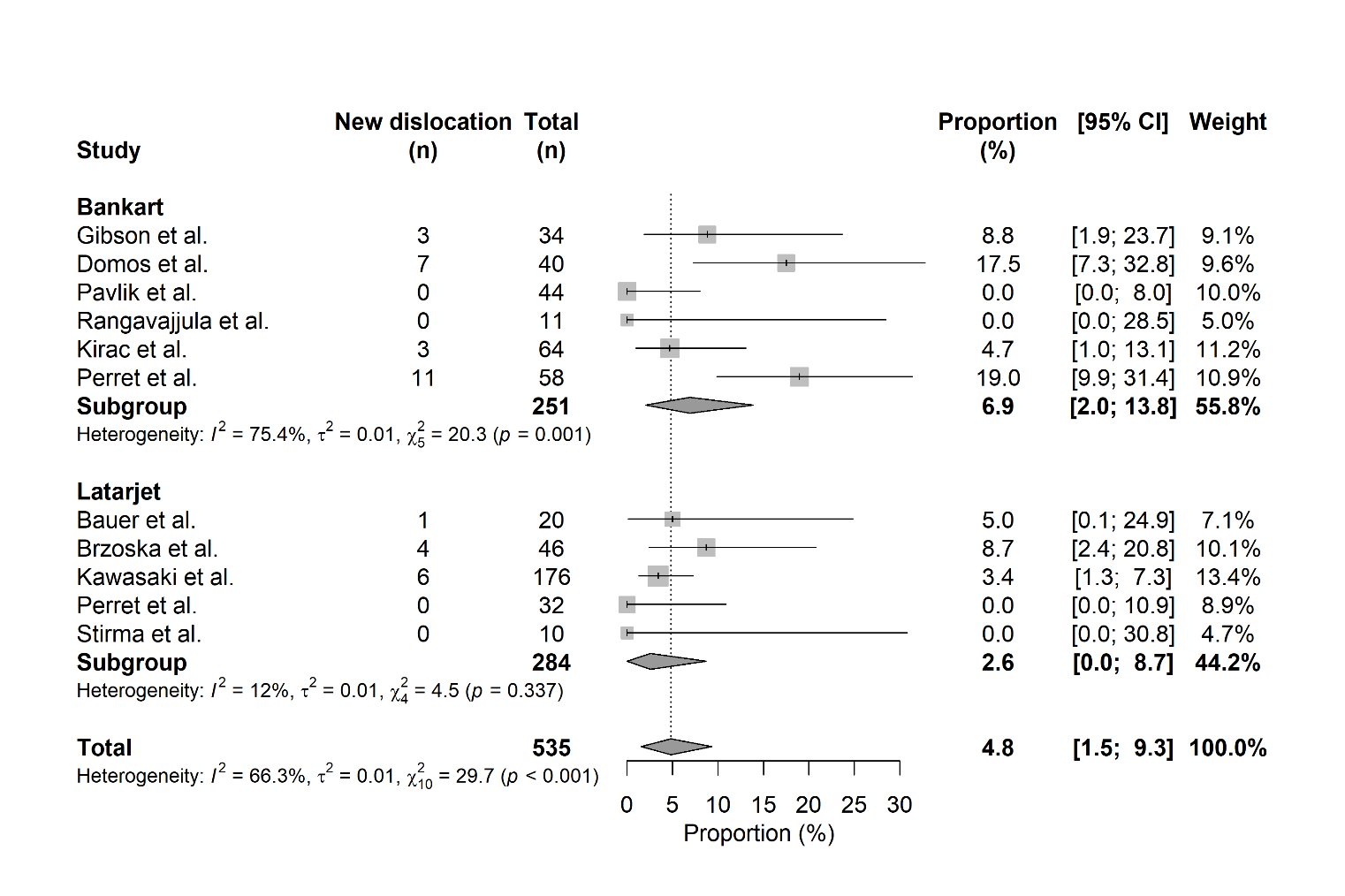
**


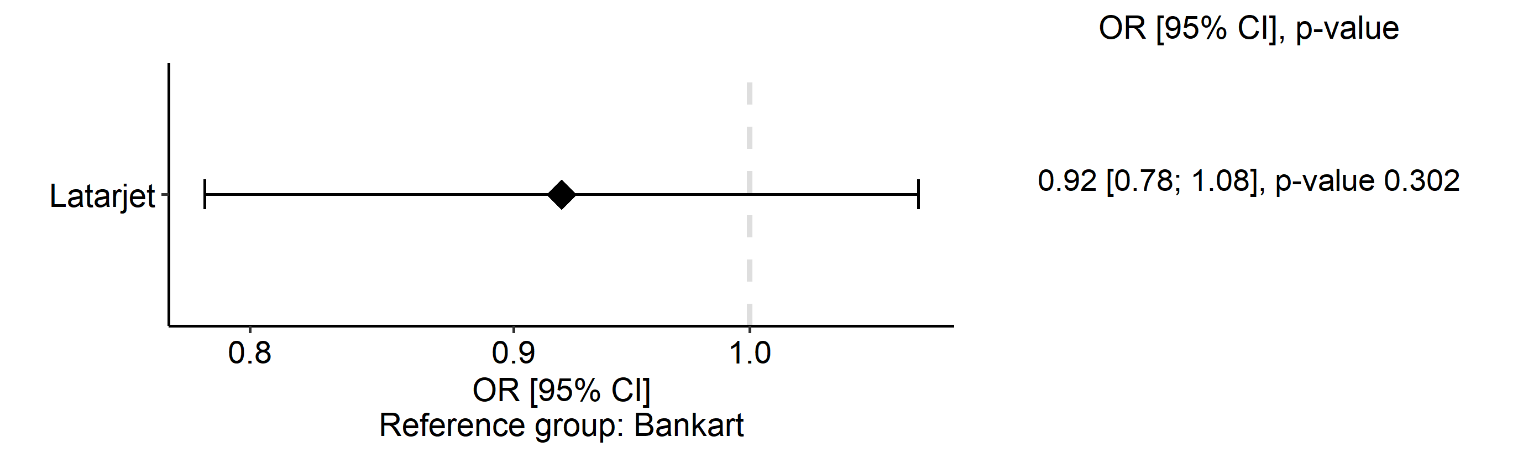


## 5.3 Funnel plot & doi plot

| Rank correlation test | p-value: 0.648 |
| --- | --- |
| Linear regression test | p-value: 0.565 |
| Doi plot | no asymmetry |
| LFK index | 0.07 |

## 5.4 Sensitivity analysis

- Overall estimate with Trim-and-fill method

Number of studies added: 0

Number of studies: 11

- Fail-safe N Calculation Using the Rosenthal Approach

Fail-safe N: 467
